# Knockdown of Laminin gamma-3 (Lamc3) impairs motoneuron guidance in the zebrafish embryo

**DOI:** 10.12688/wellcomeopenres.12394.1

**Published:** 2017-11-16

**Authors:** Alexander M. J. Eve, James C. Smith

**Affiliations:** 1 Developmental Biology Laboratory, Francis Crick Institute, London, NW1 1AT, UK

**Keywords:** axon, guidance, migration, motoneuron, netrin, parachordal chain

## Abstract

**Background**: Previous work in the zebrafish embryo has shown that laminin γ-3 ( *lamc3*) is enriched in endothelial cells marked by expression of *fli1a*, but the role of Lamc3 has been unknown.

**Methods**: We use antisense morpholino oligonucleotides, and CRISPR/Cas9 mutagenesis of F0 embryos, to create zebrafish embryos in which *lamc3* expression is compromised. Transgenic imaging, immunofluorescence, and *in situ* hybridisation reveal that Lamc3 loss-of-function affects the development of muscle pioneers, endothelial cells, and motoneurons.

**Results**: *Lamc3* is enriched in endothelial cells during zebrafish development, but it is also expressed by other tissues. Depletion of Lamc3 by use of antisense morpholino oligonucleotides perturbs formation of the parachordal chain and subsequently the thoracic duct, but Lamc3 is not required for sprouting of the cardinal vein. F0 embryos in which *lamc3* expression is perturbed by a CRISPR/Cas9 approach also fail to form a parachordal chain, but we were unable to establish a stable *lamc3* null line. Lamc3 is dispensable for muscle pioneer specification and for the expression of *netrin-1a* in these cells. Lamc3 knockdown causes *netrin-1a* up-regulation in the neural tube and there is increased Netrin-1 protein throughout the trunk of the embryo. Axonal guidance of rostral primary motoneurons is defective in Lamc3 knockdown embryos.

**Conclusions**: We suggest that knockdown of Lamc3 perturbs migration of rostral primary motoneurons at the level of the horizontal myoseptum, indicating that laminin γ3 plays a role in motoneuron guidance.

## Introduction

The zebrafish ( *Danio rerio*) is a powerful model for studying the formation of the vascular system. It is well suited to live imaging due to its transparency and external fertilisation. Development is rapid, with functional circulation established within a day of fertilisation ( Stainier & Fishman, 1994). Zebrafish are amenable to genetic, cellular, and molecular studies, and many important features of vasculogenesis are conserved with other organisms, including mammals ( Baldessari & Mione, 2008; Jing & Zon, 2011). For these reasons, zebrafish studies have already contributed to our understanding of blood and vascular development, often with relevance for human health and disease ( Bertrand *et al*., 2010; Herbert *et al*., 2009; Wiley *et al*., 2011).

Several attempts have been made to define the transcriptomes of haematopoietic and vascular precursors in the zebrafish, including the comparison of wild type and vascular deficient cloche mutants by microarray ( Stainier *et al*., 1995; Sumanas *et al*., 2005). The development of fluorescent transgenic zebrafish lines has made it possible to use fluorescence-activated cell sorting (FACS) of dissociated embryos to achieve such comparisons. The vascular reporter line Tg( *fli1a*:egfp) has frequently been used in this approach, coupled with both microarray analysis and high throughput RNA sequencing ( Lawson & Weinstein, 2002). Such work has demonstrated the enrichment of known blood and endothelial cell-specific transcripts such as *fli1a*, *tie1*, *scl*, *mpx*, and *gata1a* from GFP positive (GFP+) cells compared with gfp negative cells ( Cannon *et al*., 2013; Covassin *et al*., 2006). High throughput RNA sequencing has also revealed 388 novel transcripts enriched in zebrafish *fli1a* expressing cells between 26 and 28 hours post fertilisation (hpf), compared with the rest of the embryo ( Cannon *et al*., 2013). Following an unbiased morpholino knockdown approach to assess the importance of these novel transcripts in zebrafish angiogenesis, we focussed on loss-of-function analyses of the *lamc3* (ENSDARG00000060396) gene, which encodes the zebrafish laminin γ3 protein.

Laminins are secreted heterotrimeric glycoproteins comprising α, β, and γ chain subunits, with multiple isoforms that self-assemble in a coiled-coil configuration. Out of more than 60 possible combinations, 16 different laminin isoforms have been described ( Aumailley *et al*., 2005; Libby *et al*., 2000). Both mammals and zebrafish have five α chain isoforms named LAMA1-5. Humans ( *Homo sapiens*) have four laminin β chain isoforms (LAMB1-4), mice ( *Mus musculus*) have three β chain isoforms (LAMB1-3), and zebrafish have six laminin β genes: *lamb1*, *lamb1b*, *lamb2*, *lamb2l*, *lamb3*, and *lamb4*. In both zebrafish and mammals there are three γ chain isoforms (LAMC1-3) ( Domogatskaya *et al*., 2012).

Laminins form a major part of basement membranes, along with other proteins such as type IV collagens, perlecan, and nidogens. The laminins are involved in processes such as differentiation, migration, adhesion, and tissue organisation, as well as direct communication with cells through binding of cell surface receptors such as integrins and dystroglycans ( Domogatskaya *et al*., 2012; Durbeej *et al*., 1998; Hallmann *et al*., 2005). Laminins are required for many embryonic processes including development of the nervous system, muscle, and vascular system ( Miner *et al*., 1998; Relan *et al*., 1999). In angiogenesis, laminins have been implicated in vessel formation, vessel stability, and endothelial tip cell specification through integrin-mediated Notch signalling ( Hallmann *et al*., 2005; Kitajewski, 2011; Stenzel *et al*., 2011).

There are three laminin zebrafish mutants with reported defects in angiogenesis: *bashful* ( *bal*), *grumpy* ( *gup*), and *sleepy* ( *sly*), which encode the laminin α1, β1, and γ1 proteins respectively ( Pollard *et al*., 2006). The laminin γ3 chain is most similar in structure to γ1, but is less widely expressed. Laminin γ3 has been studied in murine basement membranes as a regulator of retinal neuronal guidance, but there has been no reported investigation into zebrafish Lamc3 ( Dénes *et al*., 2007; Gersdorff *et al*., 2005; Koch *et al*., 1999).

Here we use morpholino oligonucleotides and CRISPR/Cas9 mutagenesis of F0 embryos to characterise the consequences of laminin γ3 knockdown during development of the zebrafish trunk. We show that embryos lacking Lamc3 fail to form the parachordal chain (PAC) vasculature and that loss of the PAC is an indirect effect, caused by the failure of rostral primary motoneurons to extend at the level of the horizontal myoseptum ( Lim *et al*., 2011). This failure might be explained by the increased expression and mis-localisation of Netrin-1 observed in Lamc3 knockdown embryos.

## Methods

### Ethics statement

All zebrafish work was carried out with approval from the Francis Crick Institute Biological Research Facility Strategic Oversight Committee and the Animal Welfare and Ethical Review Body, and in accordance with the Animals (Scientific Procedures) Act 1986, the Animal Welfare Act (2006) and the Welfare of Animals in Transport Order. Care was taken to minimize the number of animals used in these experiments, in accordance with the ARRIVE guidelines.

### Protein alignments and phylogenetic trees

LAMC1 and LAMC3 amino acid sequences for human ( *Homo sapiens*), mouse ( *Mus musculus*), and zebrafish ( *Danio rerio*) were aligned using MUSCLE. Full length alignments were represented as a barcode with RasMol colouring using Geneious software ( Kearse *et al*., 2012). For phylogenetic trees, the Lamc1-like protein from *Nematostella vectensis* was included as an out-group for the bilaterians and LAMC2 sequences for human, mouse and zebrafish are included (Supplementary data repository, File 1). Aligned sequences were trimmed by eye (Supplementary data repository, File 2) within JalView software and were used for generating a JTT+Gamma model phylogenetic tree using the CIPRES gateway RaxML tool with rapid (100) bootstrapping ( Miller *et al*., 2010; Waterhouse *et al*., 2009). The generated tree was then presented using FigTree version 1.4.3 and labelled using Adobe Illustrator ( Rambaut, 2009).

### Quantitative RT-PCR

cDNA from 26 hpf FACS sorted Tg( *fli1a*:egfp) embryos was a kind gift from Elsie Place (Francis Crick Institute, London, UK), extracted as previously described ( Cannon *et al*., 2013). Quantitative RT-PCR was performed in duplicate using 10 μl reactions containing 2.5 μl of cDNA (diluted 1:10), 1× SYBR green Mastermix (Roche), and the following primers at 0.5 mM: Lamc3 Fw: 5′-GAGAACCTCTGCCACTCAGG-3′; Lamc3 Rv: 5′-CTCAAGGTGAAACGTCCCAT-3′; ef1α Fw: 5′-AAGCCCTCAGTGGAGAATGC-3′; ef1α Rv: 5′-TTGGCATCTTTAGCCACCGT-3′. Samples were run on a Lightcycler LC480 (Roche) according to the manufacturer’s instructions and expression levels were compared to a standard curve. *Lamc3* expression values were normalised to *ef1α* expression and raw qRT-PCR data (Cp-values) are located in Supplementary data repository, File 3.

### Graphs and statistical analyses

Tukey box blots and bar graphs were generated using R and annotated in Adobe Illustrator. Four statistical analyses were performed in this work: Unpaired Student’s t test was performed using Prism software to compare quantitative Lamc3 SBMO data to standard control embryos, and to generate p-values for qPCR of Lamc3 in Tg( *fli1a*:egfp) GFP+ and GFP- cells. One-way ANOVA test for multiple comparisons were performed using Prism software to generate p-values between control, uninjected, Lamc3 SBMO, and TBMO PAC quantifications. Two-way ANOVA tests were performed using Prism software to compare the number of arterial and venous intersegmental vessels between control and Lamc3 SBMO embryos. Fisher’s exact Two-tailed tests (2x2 contingency table) were performed using GraphPad software to compare the number of CRISPR/Cas9 F0 embryos with normal PAC development or PAC defects to control embryos. All raw quantified data can be found in Supplementary data repository, File 4– File 8.

### Zebrafish strains and maintenance

Zebrafish ( *Danio rerio*) adults were maintained and bred under standard conditions ( Nusslein-Volhard & Dahm, 2002). Embryos and larvae were maintained in E3 medium at 28.5°C and staged according to Kimmel *et al.*, 1995. Wild type Lon AB lines were provided by the Francis Crick Institute aquatics facility (London, UK). The transgenic line Tg( *fli1a*:egfp *, gata1*:dsRed *) ^y1^* “Tg( *fli1a*:egfp *)*” was a gift from Dr Tim Chico (MRC Centre for Developmental and Biomedical Genetics, University of Sheffield) ( Lawson & Weinstein, 2002). The Tg( *flt1*:yfp) ^*hu4624Tg*^ “Tg( *flt1*:yfp)” and Tg( *kdrl*:Hsa.HRAS-mCherry) ^*s896*^ “Tg( *kdrl*:mCherry)” lines were gifts from Dr Roger Patient (Radcliffe Department of Medicine, University of Oxford, UK) ( Chi *et al*., 2008; Hogan *et al*., 2009). The Tg( *prox1aBAC*:KalTA4-4xUAS-E1b:uncTagRFP) ^*nim5*^ “Tg( *prox1a*:rfp)” line was a gift from Dr Elke Ober (The Danish Stem Cell Center, University of Copenhagen, Denmark) ( Dunworth *et al*., 2014). The Tg( *gfap*:gfp) ^*mi2001*^“Tg( *gfap:*gfp)” transgenic line was a gift from Dr David Wilkinson (Francis Crick Institute, London, UK) ( Bernardos & Raymond, 2006).

### Probe synthesis and *in situ* hybridisation

Antisense DIG-labelled probes for Lamc3 mRNA were synthesised using amplified cDNA product using the following primers, with T7 promoter sequence underlined and SP6 sequence shown in italics: Lamc3 ISH Fw: 5′-TAATACGACTCACTATAGGGAGAGGATTCCTCTGCACTTCACTCTCT-3′; Lamc3 ISH Rv: 5′-ATTTAGGTGACACTATAGAAGNGTTGGCAGAATGAGCAGTCGCTCGTGC-3′. DIG-labelled mRNA was synthesised using 2 μg of PCR product, purified using columns according to manufacturer’s instructions (28106, Qiagen), and SP6 RNA polymerase (11487671001, Roche) in 1× transcription buffer and DIG RNA Labeling Mix (11277073910, Roche). The zebrafish *netrin-1a* ( *ntn1a*) plasmid (IRBOp991E0176D, Source Bioscience) was digested with EcoRV (R0195, NEB) according to the manufacturer’s instructions. DIG-labelled mRNA was synthesised using 1 μg of linearised plasmid DNA and T7 RNA polymerase (10881775001, Roche) in 1× transcription buffer and DIG RNA Labeling Mix (11277073910, Roche). Probes were purified using Illustra Microspin G-50 columns (27-5330-01, GE Healthcare) according to the manufacturer’s instructions. *In situ* hybridisation for *lamc3* and *ntn1a* were performed as described previously ( Thisse & Thisse, 2008). After fixation, *in situ* hybridisation expression patterns were imaged on a Leica M165FC fluorescent microscope using Leica Application Suite 3.4.1 software. Images were cropped and brightness was adjusted using Adobe Photoshop.

### Immunostaining, antibodies, and imaging

For immunostaining, all steps were performed at room temperature unless otherwise stated. Control and knockdown zebrafish embryos were fixed in 4% paraformaldehyde for 1 hour before being washed 3× in phosphate buffered saline pH7.4 (PBS) for 5 minutes each. Embryos were digested in 10 μg/ml proteinase K (AM2546, Ambion) in blocking solution (0.3% bovine serum albumin, 10% foetal calf serum, 0.2% Triton-X in PBS) for 30 minutes and refixed in 4% paraformaldehyde for 30 minutes. Next, embryos were washed 3× in PBS 0.1% Tween-20 (PBST) for 5 minutes each. Embryos were then incubated in blocking solution for 1 hour before being incubated in blocking solution with primary antibody overnight at 4°C. The next day embryos were washed 3× in PBS with 0.2% Triton-X for 10 minutes each before being incubated in blocking solution containing secondary antibody for at least 1 hour. Embryos were then washed 3× in PBS with 0.1% Triton-X for 1 hour each before a single 10 minute wash in PBST. For imaging, embryos were transferred to 75% glycerol overnight and mounted for imaging on glass cover slides. For transverse sections, embryos were cut by hand using a clean scalpel at the level of the yolk extension and mounted on glass slides with coverslips.

The following primary antibodies were used at a concentration of 1:200: rabbit anti-human LAMC3 C-terminal polyclonal primary antibody (SAB4500081, Sigma-Aldrich); rabbit anti-human NETRIN-1 (H104) polyclonal primary antibody (sc-293197, Santa Cruz Biotechnology); and mouse anti-chicken MNR/HB9/MNX1 monoclonal primary antibody (81.5C10, Developmental Studies Hybridoma Bank). Each of these antibodies was visualised using Alexa Fluor 488-conjugated goat anti-rabbit secondary (A11034, Invitrogen, Molecular Probes) used at 1:1000. The mouse anti- *Drosophila* engrailed monoclonal primary antibody (4D9, Developmental Studies Hybridoma Bank) was used at a concentration of 1:4, followed by rabbit anti-mouse HRP secondary (61-6520, Thermo Fisher Scientific) at 1:100 and Cyanine 5 tyramide reaction (SAT705A001EA, Perkin Elmer) according to the manufacturer’s instructions. In all cases, samples containing no primary antibody were used as negative controls. Whole embryos or sections were imaged on a Zeiss LSM710 confocal microscope using ZEN software. Z-stacks were compiled using FIJI (Fiji Is Just Image-J) software using a Max Intensity projection.

### Image manipulation

Where necessary for clarity, contrast was increased using Adobe Photoshop “Brightness/contrast” or “Curves” tools. All images were positioned to have anterior to the left and posterior to the right. Examples of such manipulated images compared to original images are shown in Supplementary Figure 1.

### Morpholino oligonucleotide (MO) and mRNA injection

One-cell stage embryos were injected with 1 nl solution containing up to 10 ng of antisense morpholino oligonucleotides, from Gene Tools (Philomath, OR, USA). Morpholino sequences and concentrations are shown in Table 1.

**Table 1.** Sequences for morpholino oligonucleotides.

Morpholino	Approximate concentration per embryo	Sequence (5′-3′)
Std control	10 ng	CCTCTTACCTCAGTTACAATTTATA
p53	2.5 ng	GCGCCATTGCTTTGCAAGAATTG
Lamc3 *e1i1*	5 ng	AGCCCAGTAGGGAGTCTTACCAAGA
Lamc3 translation- blocking	5 ng	AGTGAAGTGCAGAGGAATCCATCCT

For overexpression of mouse Lamc3 mRNA, the m *Lamc3* vector (IRAVp968C12150D, Source Bioscience) was linearised with NotI (R0189, NEB) according to manufacturer’s recommendations. Column purified plasmid was used as a template for *in vitro* mRNA synthesis using mMESSAGE mMACHINE SP6 transcription kit (AM1340, Ambion) according to manufacturer’s instructions. The resulting mRNA was then lithium chloride precipitated, dissolved in RNase-free water and stored in aliquots at -80°C. Either 1 nl of 200 ng/μl or 400 ng/μl of mLamc3 mRNA was injected into single embryos at the one-cell stage.

### Confocal imaging and image processing

Live embryos were transferred to a glass cover slip bottomed dish in embryo medium and 2.1 ml of stock tricaine solution (MS222, 4 mg/ml) was added per 50 ml of embryo medium. Anaesthetised animals were imaged on the Zeiss LSM710 confocal microscope using ZEN software. All images of the trunk were taken at the level of the horizontal myoseptum. 8–12 Z-stacks for images of the whole trunk, or 4–6 Z-stacks for images from the embryo midline to the distal surface of the embryo were collected at approximately 8 μm intervals. Z-stacks were compiled using Fiji (Fiji Is Just Image-J) software using “Max Intensity” projections.

### CRISPR/Cas9 mutagenesis and imaging

Custom CRISPR short guide RNAs (sgRNAs) targeting exon 1 of *lamc3* were designed using CRISPR Design tool (crispr.mit.edu) to span an endonuclease target site and constructed using the oligonucleotides shown in Table 2 generated by ZiFiT Targeter ( Sander *et al*., 2010). 100 μM oligonucleotides were phosphorylated and annealed using T4 Polynucleotide kinase (M0201, NEB) in ligation buffer for 30 mins at 37°C, 95°C for 5 minutes, and cooled to room temperature over two hours. The DR274 plasmid (a gift from Keith Young – Addgene plasmid #42250) was linearised by BsaI (R0535, NEB) digestion according to manufacturer’s instructions and gel purified (28706, Qiagen). Phosphorylated, annealed oligonucleotides (diluted 1:200) were ligated into the DR274 plasmid using the Quick ligation kit (M2200, NEB) and Plasmid Safe treatment (E3105K, Cambridge Bioscience). Template DNA was amplified from DR274 using Phusion High Fidelity polymerase (M0530, NEB) using the following primers: Fw: 5′-GCTCGATCCGCTCGCACC -3′; Rv: 5′-AAAAGCACCGACTCGGTGCC-3′; according to the manufacturer’s instructions. PCR products were gel purified (28706, Qiagen) and sgRNAs were transcribed using T7 MegaShortScript transcription kits (AM1354, Ambion) and purified using RNeasy Mini kits (74104, Qiagen) according to the manufacturers’ instructions. sgRNA quality was checked by running on a 2% agarose gel, quantified using a Nanodrop spectrophotometer and stored in aliquots at -80°C. Cas9 mRNA was synthesised from the MLM3613 plasmid (a gift from Keith Young – Addgene #42251) as described previously ( Hwang *et al*., 2013). Cas9 mRNA was purified by lithium chloride precipitation and stored in aliquots at -80°C. Two clutches of Tg( *fli1a*:egfp) zebrafish embryos were injected at the one-cell stage with 1 nl of solution containing ~12–15 ng/μl of each Lamc3 sgRNA and ~300 ng/μl of Cas9 mRNA. In addition, sgRNA alone or Cas9 mRNA alone were injected as negative controls. Embryos were imaged at 2 dpf on a Leica M165FC fluorescent microscope using Leica Application Suite 3.4.1 software. Embryos that lacked a parachordal chain in more than 5 segments of the trunk at the level of the yolk extension were recorded as having parachordal chain defects.

**Table 2.** Sequences for sgRNA oligonucleotides.

Oligonucleotide for sgRNA synthesis	Sequence (5′-3′)
Lamc3 sgRNA1 Oligo 1	TAGGACTACTGCATGCAGAC
Lamc3 sgRNA1 Oligo 2	AAACGTCTGCATGCAGTAGT
Lamc3 sgRNA2 Oligo 1	TAGGCGATTTAGCTCAGAGTCGA
Lamc3 sgRNA2 Oligo 2	AAACTCGACTCTGAGCTAAATCG

### Genotyping

To extract genomic DNA, single 48 hpf embryos or tail fin clips of individual adult fish were incubated for up to 3 hours at 55°C in 20–50 μl of genomic DNA extraction buffer (50 mM Tris-HCl pH 8.5, 1 mM EDTA, 0.5% Tween-20) with fresh 0.08 μg/ml Proteinase K (AM256, Ambion) added before use. Samples were then heated to 95°C for 10 minutes to inactivate proteinase K and debris was spun down using micro centrifugation. The sgRNA target site was amplified from extracted genomic DNA using the following primers: Fw: 5′-TTCTGCTTTTTGCCAGCGTC-3′; Rv: 5′-GCAATACCAGCACTGCTCTAC-3′. Mutation of the target site was identified by digestion with SphI-HF (R3182, NEB) or TaqαI (R0149, NEB) for sgRNA1 and sgRNA2 respectively, overnight at 37°C according to the manufacturer’s instructions. After digestion, samples were run on a 2% agarose gel for 30 minutes at 120V and compared to a 100 bp ladder (15628-050, Life Technologies). For SphI digests, complete digestion of wild type product produces three bands of 84, 164 and 263 bp. Some animals were later identified to contain a single nucleotide polymorphism (SNP) in one of the SphI sites. Complete digestion of wild type genomic DNA in such animals produces two bands of 164 and 346 bp. Digestion of mutant product yields bands of 83 and 427 bp, or 510 bp if the SNP is present. For TaqαI digests, complete digestion of wild type PCR product produces two bands of 205 and 305 bp. Digestion of mutant products yields a single band of 510 bp. For presentation, agarose gel images were inverted and cropped but were not otherwise manipulated.

### Protein extraction and western blot

48 hpf zebrafish embryos were dechorionated in batches of around 100 and transferred to ice cold Calcium-free Ringer’s solution. Embryos were rinsed 3× in Ringer’s solution. Samples were then transferred to ice cold Ringer’s solution containing 1mM EDTA and 0.3 mM phenylmethylsulfonyl fluoride (PMSF). Embryos were de-yolked by pipetting using a 200 μl pipette tip. De-yolked embryos were then rinsed twice in Ringer’s solution with EDTA and PMSF, and snap frozen on dry ice. Frozen embryos were thawed and homogenised in 100 μl of protein extraction buffer (1% IGEPAL, 150 mM NaCl, 20 mM Tris pH 7.5, 2 mM EDTA, 50 mM NaF, 1mM sodium pyrophosphate) with 1× cOmplete Mini Protease Inhibitor Cocktail (1183615300, Roche) overnight at 4°C with rotation. Samples were pelleted and 150 μl of 1× Laemmli sample buffer was added (161-0737, BioRad). Pellets were homogenised in sample buffer using a microfuge pestle in a round-bottom 1.5 ml tube. Before loading, samples were boiled at 99°C for 5 minutes.

20 μl of protein extract was loaded into a NuSEP 4–20% glycine pre-cast gel with Spectra multicolour broad range protein ladder (SM1849, Thermo Scientific) and run in SDS running buffer for 1 hour at 100V. Proteins were transferred to methanol-activated Immobilon-P PVDF membranes (Millipore) in Tris-Glycine transfer buffer. Transfer was performed for approximately 1 hour at 400 amps with ice cooling. Membranes were blocked in 5% milk powder in PBST (phosphate buffered saline pH7.4, 0.1% Tween-20) for 1 hour at room temperature. Membranes were incubated with rabbit anti-human NETRIN-1 (H104) polyclonal antibody (sc-293197, Santa Cruz Biotechnology) was in 5% milk powder blocking solution. For data presented in this work, the antibody was used at a concentration of 1:100, although bands of identical molecular weights can also be detected up to a concentration of 1:1000. Membranes were rinsed with 5 washes in PBST before being incubated with goat anti-rabbit HRP-conjugated antibody (31460, Thermo Scientific) 1:2000 in 5% milk powder blocking solution for 2 hours at room temperature. Membranes were rinsed 3× in PBST at room temperature. Protein bands were visualised using chemifluorescence, SuperSignal West Dura Extended Duration Substrate (34075, Thermo Scientific) according to manufacturer’s instructions and exposed using the ChemiDoc gel imaging system (BioRad). An image of the gel prior to cropping and brightness/contrast manipulation can be found in the Supplementary data repository, File 9. Molecular weights were compared to the protein ladder and putatively identified according to predicted molecular weights ( Table 3).

**Table 3.** Predicted molecular weights of zebrafish Netrin-1 proteins using ExPASy Compute pI/MW tool. aa, amino acids; kDa, kilo Daltons; MW, molecular weight.

ENSEMBL Transcript ID	Name	Uniprot code	Size (aa)	Predicted MW (kDa)
ENSDART00000157527.1	Nertrin-1a	O42140_DANRE	603	65307.06
ENSDART00000171384.1	Nertrin-1a	A0A0R4IN40_DANRE	446	48191.08
ENSDART00000039482.4	Nertrin-1b	F1QUK5_DANRE	602	65338.19

## Results

### Lamc3 is expressed by endothelial cells

**Figure 1.**
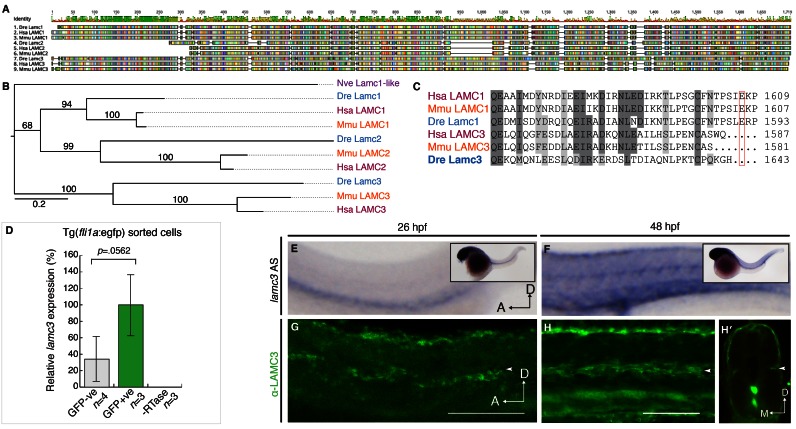
*Lamc3* expression during zebrafish development. ( **A**) MUSCLE-aligned protein “fingerprints” of zebrafish ( *Danio rerio*, Dre), human ( *Homo sapiens*, Hsa), and mouse ( *Mus musculus*, Mmu) LAMC1, LAMC2, and LAMC3 amino acid sequences with RasMol colouring. ( **B**) Unrooted RaxML phylogenetic tree (JTT+Gamma model) of *Nematostella vectensis* (purple, Nve) Lamc1-like and human (red, Hsa), mouse (orange, Mmu), and zebrafish (blue, Dre) LAMC1, LAMC2, and LAMC3 proteins. Branch labels denote ML bootstrap values (100). ( **C**) Aligned human, mouse, and zebrafish amino acid sequence of the laminin γ1 and γ3 C-terminal tails show that the loss of the essential glutamic acid residue in γ3 is conserved across vertebrates. ( **D**) qRT-PCR shows *lamc3* is not significantly enriched in *gfp*+ endothelial cells (1.85E-05±5.49E-06, n=3) compared to GFP- cells (6.28E-06±1.48E-06, n=4) sorted from 26 hpf Tg( *fli1a*:egfp) embryos. Expression is relative to *ef1α* expression and normalised to GFP+ population (100%). All values are mean±s.e.m. P-value determined by Unpaired Two-tailed Student T-test. ( **E**– **F**) Whole-mount *in situ* hybridisation showing *lamc3* expression in the whole embryo and enlarged lateral view of the trunk as indicated. ( **E**) At 26 hpf *lamc3* is expressed in the head vasculature and in major trunk vasculature (n=10). ( **F**) At 48 hpf *lamc3* is also expressed in myotomes at the level of the horizontal myoseptum (n=15). ( **G**– **H**) Immunostaining using an anti-LAMC3 C-terminal antibody. ( **H**) At 26 hpf Lamc3 protein is localised in the dorsal region of the trunk and at the level of the HMS (arrowhead, n=10). (I) By 48 hpf protein is also detected ventrally in the region of the dorsal aorta and posterior cardinal vein (n=5). A, anterior; D, dorsal; dpf, days post fertilisation; hpf, hours post fertilisation; n, number of cDNA samples. Scale bars: 100 μm.

Amino acid sequences of human, mouse, and zebrafish laminin γ1, γ2, and γ3 chains were aligned using MUSCLE ( Figure 1A). Zebrafish laminin γ3 is a large protein (1643 amino acids) with several annotated domains including a laminin-binding domain at the N-terminus, 11 epidermal growth factor-like (EGF-like) binding domains, a laminin IV (L4) domain (the function of which is unknown), and a C-terminal coiled-coil domain ( Domogatskaya *et al*., 2012). Phylogenetic analysis of human, mouse, and zebrafish LAMC1, LAMC2, and LAMC3 proteins shows that LAMC3 is most closely related to LAMC1 ( Figure 1B). LAMC3 is unable to bind integrins because it lacks an essential glutamic acid residue in the C-terminal tail ( Ido *et al*., 2008). Amino acid alignments showed that this is conserved between vertebrates ( Figure 1C).

High-throughput sequencing of sorted 26–28 hpf Tg( *fli1a*:egfp) zebrafish embryos had suggested that *lamc3* is enriched 4-fold in *fli1a*:gfp expressing (GFP+) endothelial cells ( Cannon *et al*., 2013). To verify this observation we performed qRT-PCR on cDNA synthesised from FACS sorted 26 hpf Tg( *fli1a*:egfp) embryos. Our results revealed only a 3-fold enrichment that was not statistically significant (p=0.0562, Unpaired Student’s t-test) in GFP+ cells compared with the rest of the embryo. This observation confirms that *lamc3* is expressed by GFP+ cells, but suggests that other cells also express the gene ( Figure 1D). *Fli1a* is also expressed in the pharyngeal arch and neural crest ( Lawson & Weinstein, 2002). Therefore, to determine whether the Lamc3 detected in GFP+ cells derives from endothelial cells, we investigated the spatial expression of *lamc3* by whole-mount *in situ* hybridisation. In 26 hpf embryos, *lamc3* is expressed in the head and vasculature (n=10) ( Figure 1E). In addition, expression is seen in the fin bud and myotome at the level of the horizontal myoseptum at 48 hpf (n=15) ( Figure 1F). These data are consistent with published *in situ* hybridisation patterns and suggest that *lamc3* is expressed in both vascular endothelium and non-endothelial cells, including the middle cerebral vein, dorsal neuronal plate, and gut ( Sztal *et al*., 2011).

Laminins are secreted proteins, so we used whole-mount immunostaining to ask whether laminin γ3 protein (γ3) is found adjacent to regions of gene expression ( Figure 1G–H). At 26 hpf, low levels of γ3 were detected in the dorsal floor plate of the neural tube and the horizontal myoseptum, but no protein was observed in the trunk vasculature (n=10). At 48 hpf (n=5) γ3 protein overlaps with *lamc3* expression: protein is located at the horizontal myoseptum, the dorsal plate of the neural tube, and axial vessels in the trunk ( Figure 1H′). To show that this was specific to γ3, we overexpressed mouse Lamc3 mRNA in zebrafish embryos and detected a dose-dependent increase in labelling in these regions at 24 hpf ( Figure S2 A–C). We believe that overexpressed protein accumulates in regions of endogenous expression because suitable laminin alpha and beta chains need to be co-expressed to form a heterotrimeric complex.

### Lamc3 knockdown embryos have depleted γ3 protein

**Figure 2.**
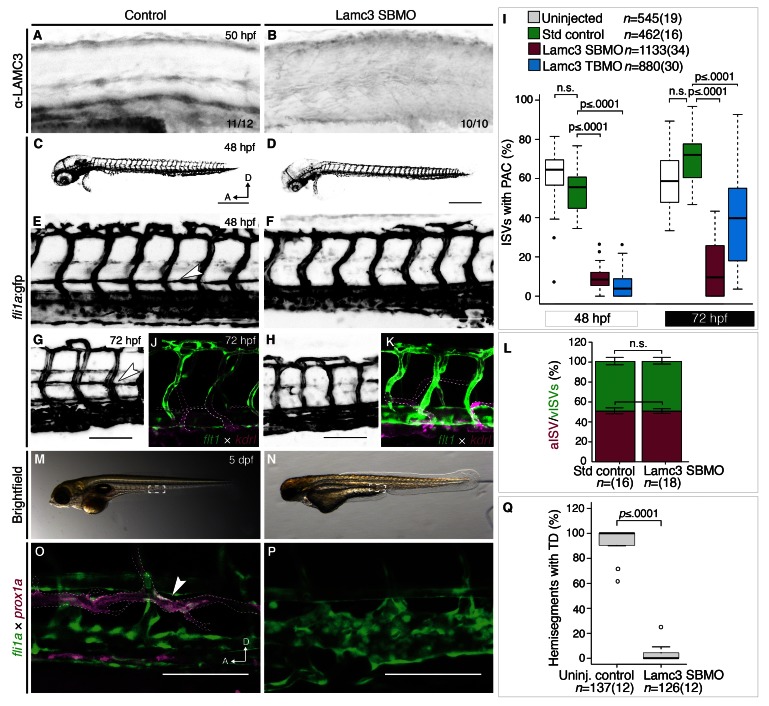
Lamc3 knockdown embryos do not develop the parachordal chain (PAC). ( **A**– **B**) Lateral view of the zebrafish trunk stained using anti-LAMC3 antibody shows reduction in Lamc3 protein at 50 hpf in knockdown embryos ( **E**, n=10) compared to standard controls ( **D**, n=11). ( **C**– **I**) Vasculature of 48 hpf Tg( *fli1a*:egfp) embryos. ( **C**) Embryos injected with standard control MO develop the PAC. ( **D**) Lamc3 knockdown embryos do not develop the PAC. ( **E**) An enlarged image of the trunk vasculature in control embryos the PAC is indicated by a white arrowhead. ( **F**) An enlarged image of the trunk vasculature in Lamc3 SBMO injected embryos. ( **G**) The parachordal chain in control embryos at 72 hpf (white arrowhead). ( **H**) By 72 hpf Lamc3 embryos have still not developed a parachordal chain. ( **I**) Quantification of all ISVs with a PAC in Tg( *fli1a*:egfp) uninjected (white, n=545), control (green, n=462), Lamc3 SBMO knockdown (magenta, n=1133) and Lamc3 TBMO knockdown embryos (blue, n=880) at 48 hpf and 72 hpf, represented as a Tukey box plot. P-value determined by One-way ANOVA test for multiple comparisons. ( **J**– **L**) Venous sprouting is not affected by Lamc3 knockdown. ( **J**, **K**) Lateral view of the trunk of Tg( *flt1*:yfp; *kdrl*:mCherry) embryos show venous intersegmental vessels (vISVs, magenta, outlined) and arterial ISVs (aISVs, green/white) in both control (n=30) and knockdown embryos (n=34). ( **L**) Quantification of the number of aISVs (green) and vISVs (magenta) shows no significant difference between controls (n=16) and Lamc3 SBMO injected embryos (n=18). P-value determined by Two-way ANOVA test. ( **M**– **Q**) Lamc3 knockdown embryos lack the thoracic duct. Brightfield view of 5 dpf embryos shows developmental delay in Lamc3 knockdown embryos ( **N**, n=40) compared to controls ( **M**, n=35). Lateral view of the trunk of Tg( *fli1a*:egfp; *prox1a*:rfp) embryos shows TD (magenta, arrowhead, outlined) in control embryos ( **O**, n=10) is lost in Lamc3 knockdown embryos ( **P**, n=9). ( **Q**) Quantification of TD per 10 consecutive hemisegments in Tg( *kdrl*:egfp; *prox1a*:rfp) uninjected (n=12) or Lamc3 SBMO knockdown embryos (n=12) at 5 dpf shown in a Tukey box plot. P-value determined by Unpaired Student’s t test comparing TD. A, anterior; d, days post fertilisation; D, dorsal; hpf, hours post fertilisation; n, number of hemisegments (number of embryos); n.s., not significant (p>0.05). Scale bars: 100 μm.

To validate Lamc3 knockdown, embryos injected with standard control morpholino or Lamc3 SBMO were immunostained using the human anti-LAMC3 C-terminal antibody. Control embryos (n=11/12) showed γ3 protein in the dorsal plate of the neural tube, horizontal myoseptum and ventral vasculature as seen previously ( Figure 2A). In Lamc3 knockdown embryos (n=10) we observed a reduction in γ3 protein in the vasculature and horizontal myoseptum although it is possible that some protein persists in the dorsal neural plate ( Figure 2B).

### Lamc3 is required for parachordal chain (PAC) formation

Lamc3 is expressed in fli1a+ endothelial cells, so we asked whether γ3 is required for zebrafish vascular development. To this end, we injected translation blocking (TBMO) and splice-blocking morpholino oligonucleotides (SBMO) targeting Lamc3 mRNA into Tg( *fli1a*:egfp) embryos and observed the developing vasculature over the following 3 days. A minority of MO injected embryos exhibited phenotypes associated with MO toxicity, including cell death and oedema in the head ( Figure S3 A,B) ( Eisen & Smith, 2008). For this reason, in the experiments that follow we co-injected MOs with p53 MO to control for off-target effects.

Our results show that in uninjected (n=19) or standard control morpholino injected embryos (n=16) the parachordal chain (PAC, white arrowhead) develops between the intersegmental vessels at the level of the horizontal myoseptum ( Figure 2C,E). In embryos injected with Lamc3 SBMO (n=34) and TBMO (n=30) the PAC fails to develop ( Figure 2D,F). To account for any developmental delay causing the PAC defect, control or Lamc3 knockdown embryos were allowed to develop until 72 hpf. At 72 hpf Lamc3 SBMO injected embryos (n=38) recovered development of the dorsal longitudinal anastomotic vessel (DLAV) but the PAC remained absent ( Figure 2G,H). In Lamc3 TBMO embryos (n=16) the common cardinal vein had enclosed the perimeter of the yolk, but the embryos continued to lack a PAC ( Figure S3C). These data suggest that the defects in PAC development are not a result of general developmental delay. Quantification of the number of PACs per intersegmental vessel determined that Lamc3 knockdown embryos had significantly fewer (p<0.0001, One-way ANOVA test for multiple comparisons) PACs compared to controls at both time points ( Figure 2I). This phenotype persisted longer using the Lamc3 SBMO, and this MO was therefore used in all subsequent experiments.

Loss of the PAC in embryos lacking γ3 could result from failure of the venous intersegmental vessels to sprout dorsally from the posterior cardinal vein ( Hogan *et al*., 2009). To determine if Lamc3 knockdown prevents venous sprouting, Tg( *flt1*:yfp; *kdrl*:mCherry) embryos were injected with Lamc3 SBMO and venous intersegmental vessels were identified by their lack of flt1 expression, which is specific to arterial endothelium ( Swift & Weinstein, 2009). At 72 hpf, both standard control (n=16) embryos have vessels that lack *flt1* expression (white outline), which form the PAC and venous intersegmental vessels ( Figure 2J). Laminin γ3-depleted embryos (n=18) also showed intersegmental vessels of venous origin (white outline) that did not express *flt1*:yfp ( Figure 2K). Numbers of arterial (p=0.9718) and venous (p=0.5326) intersegmental vessels were counted and were not significantly different (Two-way ANOVA test) between control and Lamc3 knockdown embryos ( Figure 2L). This suggests that Lamc3 is not required for sprouting from the cardinal vein.

### Lamc3 knockdown embryos do not develop a thoracic duct

The parachordal chain is the source of lymphatic endothelial cells that form the thoracic duct ( Okuda *et al*., 2012). Therefore, we asked whether thoracic duct development is affected by Lamc3 knockdown. Tg( *fli1a*:egfp; *prox1a*:rfp) embryos were injected with Lamc3 SBMO (n=12) or standard control MO (n=12) and were observed at 5 dpf for the presence or absence of the thoracic duct, identified by expression of *prox1a*. Despite increasing in size, γ3-deficient embryos were severely morphologically abnormal compared with controls ( Figure 2M,N). In control embryos (n=12) the thoracic duct (white outline, white arrowhead) is clearly visible between the dorsal aorta and posterior cardinal vein ( Figure 2O). We observed no *prox1a*-expressing thoracic duct cells in Lamc3 SBMO injected embryos ( Figure 2P). To quantify this observation, the numbers of hemisegments with a thoracic duct were counted and γ3-deficient embryos had a significant (p<0.0001, Unpaired Student’s t test) reduction compared to controls ( Figure 2Q). It was unclear whether the lack of thoracic duct in Lamc3 SBMO embryos is a result of developmental delay, caused by an earlier developmental defect or as a direct result of γ3-deficiency.

### CRISPR/Cas9 genetically modified F0 embryos recapitulate the MO knockdown phenotype

**Figure 3.**
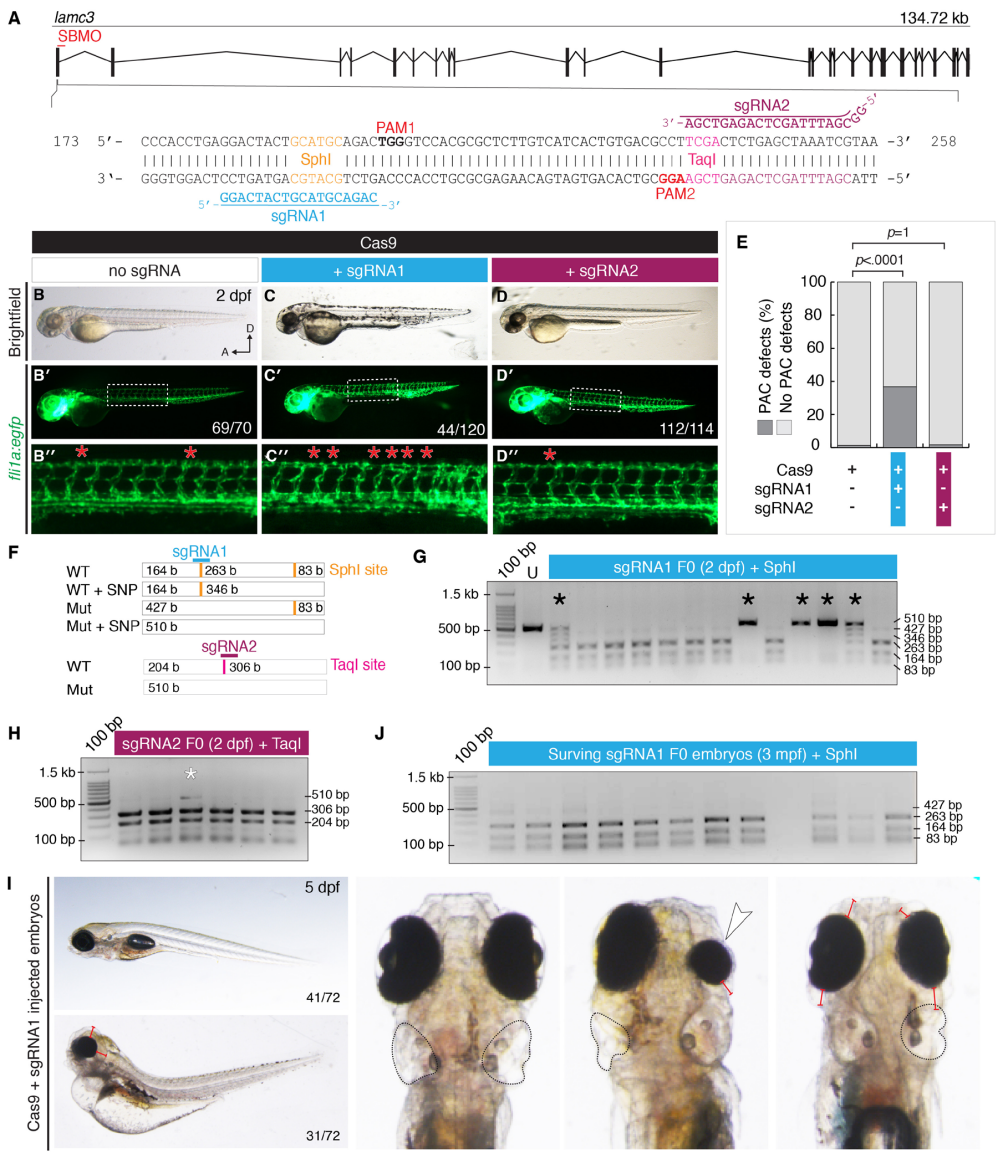
CRISPR/Cas9 mutagenesis of *lamc3* causes defects in parachordal chain (PAC) development. ( **A**) Design of sgRNAs targeted to exon1 of the *lamc3* gene. Each sgRNA targets a restriction endonuclease site used for genotyping and is upstream of a protospacer adjustment motif (PAM). ( **B**– **D**) Cas9/sgRNA injected embryos (as labelled) at 50 hpf. ( **B**– **D**) Brightfield images show no developmental defects or developmental delay. ( **B′**– **D′**) Whole embryo images of Tg( *fli1a*:egfp) fluorescence area indicated by white dotted line is enlarged in ( **B′′**– **D′′**) the PAC in each hemisegment is indicated with a red asterisk. ( **E**) Quantification of Tg( *fli1a*:egfp) embryos at 50 hpf with PAC defects. P-values determined by Fisher’s exact Two-tailed test compared to Cas9 alone controls. ( **F**– **I**) 2% agarose genotyping gels with 100 bp ladder. ( **F**) Schematic diagram of the region amplified by PCR contains two SphI sites (orange), which would digest into three fragments of 83, 164 and 263 bp in length. Predicted mutation of one of these sites will produce two bands of 83 and 427 bp in length. A natural variant contains a SNP in the second SphI site, complete digestion of this variant would produce two bands of 164 and 346 bp in length. Mutation of the target SphI site would prevent digestion of the fragment. Digestion of the amplicon with Taq ^α^I (blue) in wild type embryos will yield two fragments of 204 and 306 bp in size. ( **G**) Restriction endonuclease digest of target region shows undigested mutant products (black asterisk) at a size of 427/510 bp in sgRNA1-injected embryos. ( **H**) Fewer undigested mutant products (510 bp) were observed in sgRNA2-injected embryos. ( **I**) Brightfield images sgRNA1/Cas9 injected embryo at 5 dpf shows severe oedema. ( **J**) Genotyping of 3-month adult fish. A, anterior; b/bp, base pairs; d, days post fertilisation; D, dorsal; kb, kilobases; mpf, months post fertilisation; n, number of embryos; PAC, parachordal chain; PAM, protospacer adjustment motif; SBMO, splice-blocking morpholino; sgRNA, short guide RNA; U, undigested control;.

Recent work has suggested that antisense morpholinos may cause misleading results through off-target effects, and may not always represent a true loss-of-function phenotype ( Kok *et al*., 2015). To address this concern, we used the CRISPR/Cas9 system to genetically alter the *lamc3* gene in F0 embryos. Previous work has shown that this approach can yield biallelic mutant embryos that recapitulate morpholino knockdown phenotypes ( Jao *et al*., 2013). Two sgRNAs targeting the first exon of *lamc3* were designed ( Figure 3A). Each sgRNA was injected into 1-cell Tg( *fli1a*:egfp) embryos together with Cas9 mRNA, and Cas9 mRNA alone was also injected as a negative control. At 2 dpf embryos were assessed for formation of the PAC. Of embryos injected with Cas9 mRNA alone (n=70), only 1 had developmental defects ( Figure 3B,E). Of embryos injected with sgRNA1 and Cas9 mRNA (n=120), the majority of embryos looked morphologically normal although a small number (<5%) showed signs of toxicity such as curvature of the body axis and intersegmental vessel defects. 44 had five or more absent PACs, significantly more (p<0.0001, Fisher’s exact Two-tailed test) than those injected with Cas9 mRNA alone ( Figure 3C,E). In contrast, the majority of embryos injected with sgRNA2 and Cas9 mRNA (n=112/114) had normal PAC development ( Figure 3D,E).

Random restriction enzyme genotyping of injected single embryos showed that sgRNA1 was more effective than sgRNA2 at inducing mutations ( Figure 3F–H). We also observed biallelic mutations from injection of sgRNA1 and Cas9 mRNA ( Figure 3G). Considerably fewer mutant cells and no biallelic mutations were detected in embryos injected with sgRNA2, so these embryos might serve as a negative control for sgRNA-induced off target effects ( Figure 3H). Indeed, most embryos injected with sgRNA2 developed normally ( Figure 3D–E). To show that PAC defects were not a nonspecific result caused by introduction of short oligonucleotides into the cell, embryos were injected with sgRNA1 alone or with Cas9 mRNA. A few embryos (n=3/51) had developmental defects when Cas9 mRNA was absent ( Figure S4A), whereas significantly more (p=0.0056, Fisher’s exact two-tailed test) defects were observed in embryos with both Cas9 mRNA and sgRNA1 ( Figure S4B). These observations, together with the morpholino knockdown data, lead us to conclude that PAC development is affected by γ3-deficiency.

We attempted to generate a stable *lamc3* mutant line by injecting Tg( *fli1a*:egfp) embryos with Cas9 mRNA and sgRNA1. However, by 5 dpf almost half (n=31/72) of the injected embryos presented severe oedema, with swelling around the eyes (red lines), heart, gut and yolk ( Figure 3I). In addition, we observed partial reductions in eye size (white arrowhead) and defects in otic vesicle formation (black outline). Larvae with oedema were reproduced in subsequent injections and not kept past 5 dpf. Of the embryos that survived beyond 5 dpf, only 12 reached breeding age. Genotyping of these adults indicated little or no mutagenesis in these animals ( Figure 3J) and no heterozygous *lamc3* mutants were recovered from subsequent crosses. Because we have been unable to create a *lamc3* mutant line using CRISPR/Cas9, subsequent experiments make use of antisense morpholino oligonucleotides.

### Notochord development is unaffected in Lamc3 knockdown embryos

**Figure 4.**
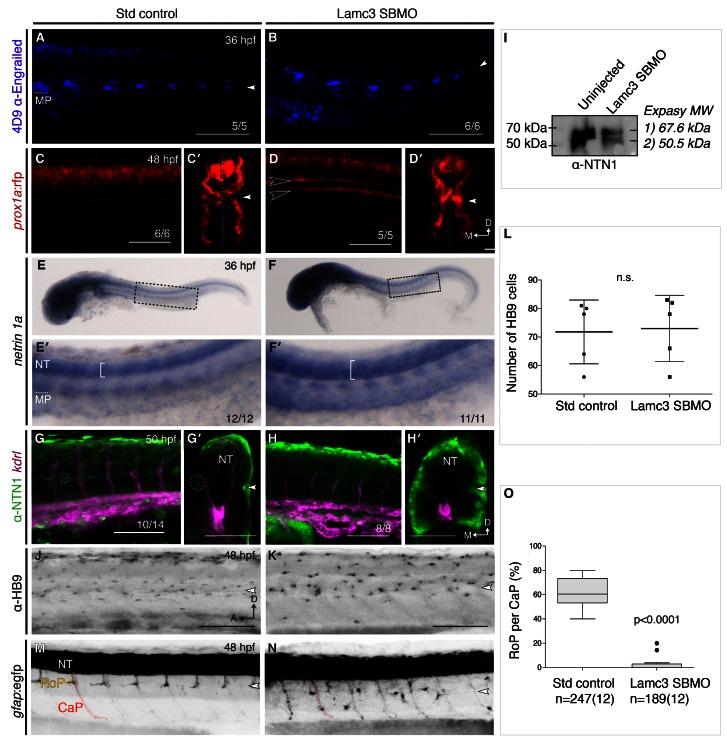
Lamc3 knockdown affects rostral primary motoneuron (RoP) migration. ( **A**– **B**) Lateral view of 36 hpf embryos stained for muscle pioneers (MPs, underlined) using 4D9 anti-engrailed show MPs present in both control ( **A**, n=5) and knockdown embryos ( **B**, n=6). ( **C**– **D**) 48 hpf Tg( *prox1a*:rfp) embryos injected with standard control ( **C**, n=6) or Lamc3 SBMO ( **D**, n=6) have increased *prox1a* expression in the trunk. Transverse sections in **E′** and **D′** show additional *prox1a* expressing cells at the horizontal myoseptum in Lamc3 SBMO embryos. ( **E**– **F**) Whole-mount *in situ* hybridisation for *netrin-1a* expression at 36 hpf. *Netrin-1a* is expressed in MPs and neural tube (NT) in both control ( **E**, n=12) and knockdown embryos ( **F**, n=11). Highlighted regions are enlarged in **E′** and **F′** and indicate an increased expression domain in the NT of knockdown embryos. ( **G**– **H**) Lateral view of the trunk of 50 hpf Tg( *kdrl*:mCherry) (magenta) embryos stained for Netrin-1 (anti-NETRIN-1, green). Loss of Netrin-1 at the horizontal myoseptum (HMS) in knockdown embryos ( **H**, n=8) compared to controls ( **G**, n=10/14). Transverse sections of the embryo trunk in **G′** and **H′** shows Netrin-1 protein is restricted to the HMS and periphery in controls, but is widely distributed through the embryo in Lamc3 knockdown embryos. ( **I**) Western blot of whole zebrafish embryo extracts using the human anti-NETRIN-1 polyclonal antibody shows two bands of molecular weights between 50–70 kDa. ( **J**– **L**) HB9 (anti-MNR2) immunostaining of motoneurons. Knockdown embryos ( **K**, n=5) show change in cell morphology and organisation compared to controls ( **J**, n=5). ( **L**) Quantification of HB9 positive cells shows no significant difference between control and knockdown embryos, represented as a scatter plot showing mean and standard deviation. P-value determined by Student’s t test. ( **M**– **O**) Tg( *gfap*:gfp) embryos injected with standard control ( **M**) or Lamc3 SBMO morpholino ( **N**). Lamc3 knockdown (n=12) showed increased branching of caudal primary motoneurons (CaP, red outline), and a loss of RoPs (yellow outline) at the horizontal myoseptum compared to controls (n=12). ( **O**) Quantification of RoPs at the horizontal myoseptum per CaP represented as a Tukey box plot. There was a significant (p<0.0001) reduction of RoPs in Lamc3 knockdown embryos. P-value determined by Student’s t test. n, number of CaPs (number of embryos). Small white arrowhead denotes the horizontal myoseptum. A, anterior; D, dorsal; CaP, caudal motoneuron; h, hours post fertilisation; M, medial; MP, muscle pioneers; NT, neural tube; RoP, rostral primary motoneurons. Scale bars: 100 μm.

Mutations in other laminins cause notochord defects that prevent proper angiogenesis ( Odenthal *et al*., 1996; Parsons *et al*., 2002; Pollard *et al*., 2006). Sonic hedgehog derived from the notochord is crucial for the specification of muscle pioneers at the horizontal myoseptum, which develop into slow-twitch muscle ( Blagden *et al*., 1997). To see if notochord development was perturbed in Lamc3 knockdown embryos we asked if muscle pioneers were induced at 36 hpf. Immunofluorescence using the 4D9 anti-Engrailed antibody in both control (n=5) and knockdown (n=6) embryos showed that muscle pioneer cells were indeed specified at the horizontal myoseptum ( Figure 4A–B). This distinguishes the role of γ3 in angiogenesis from that of other laminins that are required for notochord development ( Dolez *et al*., 2011). We then asked whether slow-twitch muscle also developed normally in Lamc3 morphants using the Tg( *prox1a*:rfp) line. In control embryos (n=6) *prox1a*:rfp was expressed in the neural tube, caudal motoneurons and slow-twitch muscle ( Figure 4C). Transverse sections showed slow-twitch muscle flanking the neural tube along the length of the embryo ( Figure 4C′). To our surprise, ectopic *prox1a*:rfp was expressed in unknown cells at the horizontal myoseptum (black arrowheads) in Lamc3 morphants ( Figure 4D′). However *prox1a*:rfp was also expressed in the somites, suggesting that slow-twitch muscle does differentiate, although it has not as migrated as far to the periphery of the embryo as in controls ( Figure 4D′).

### Netrin-1 is affected by Lamc3 knockdown

Muscle pioneers express *netrin-1a* at the horizontal myoseptum and knockdown of the zebrafish Netrin-1a and its cognate receptors: *unc5b* and *cd146* prevents formation of the PAC, phenocopying Lamc3 knockdown ( Navankasattusas *et al*., 2008; Park *et al*., 2004). Furthermore, Netrin-1 provides guidance for the axon migration of motoneurons, which are required for PAC development ( Hale *et al*., 2011; Lauderdale *et al*., 1997; Lim *et al*., 2011). To ask whether the expression of *netrin-1a* by muscle pioneers is disturbed, we performed *in situ* hybridisation on 36 hpf control and Lamc3 knockdown embryos. We found that expression of *netrin-1a* by muscle pioneer cells was not altered in Lamc3 morphants (n=11) compared with control embryos (n=12). To our surprise however, the expression domain of *netrin-1a* appeared to be broader in γ3-deficient embryos, with increased expression in the neural tube ( Figure 4E,F).

To visualise Netrin-1 protein, immunofluorescence was performed on 50 hpf control (n=10/14) and Lamc3 SBMO (n=8) injected Tg( *kdrl*:mCherry) embryos using a human anti-NETRIN-1 polyclonal antibody. This antibody was chosen because its epitope is highly conserved between human and zebrafish Netrin-1a and Netrin-1b proteins. In control embryos, we observed Netrin-1 protein in the neural tube and in the middle of somites (white outline) in the trunk ( Figure 4G). Netrin-1 protein was reduced in all Lamc3 knockdown embryos at the level of the horizontal myoseptum compared with controls ( Figure 4H). However, transverse sections showed that Netrin-1 was widely dispersed in Lamc3 knockdown embryos and that Netrin-1 protein was located throughout the majority of the trunk ( Figure 4H′). We validated the anti-NETRIN-1 antibody by Western blot using protein extracts from whole 48 hpf zebrafish embryos either uninjected or injected with Lamc3 SBMO. Results showed two bands corresponding to the predicted molecular weights of zebrafish Netrin-1 splice variants ( Figure 4I).

### Rostral primary motoneurons are affected by Lamc3 knockdown

The development of the PAC depends on the correct migration of rostral primary motoneurons, which extend axons along the horizontal myoseptum in response to Netrin-1a signalling ( Hale *et al*., 2011; Lim *et al*., 2011). *Netrin-1a* expression is perturbed in Lamc3 knockdown embryos and LAMC3 has an important role in mammalian neuronal development, so we investigated motoneuron development in γ3-deficient embryos ( Gnanaguru *et al*., 2013; Li *et al*., 2012; Tanabe *et al*., 1998). We used an anti-HB9 (MNX1) antibody and a transgenic Tg( *gfap*:gfp) zebrafish line to ask whether primary motoneurons are affected by Lamc3 knockdown at 48 hpf.

In 48 hpf control embryos (n=5) HB9 positive cells are elongated along the anterior-posterior axis and are concentrated at the dorsal neural tube and horizontal myoseptum ( Figure 4J). The motoneurons in Lamc3 knockdown embryos are found in similar regions, although they have a rounded morphology ( Figure 4K). The total number of HB9 positive motoneurons in Lamc3 knockdown embryos (73±12) did not differ significantly from controls (72±11; p>0.05, Unpaired Student’s t test) suggesting that Lamc3 is not required for specification of HB9 positive motoneurons but may be required for regulating their shape or polarity ( Figure 4L).

To study motoneurons in more detail, Lamc3 was knocked down in Tg( *gfap*:gfp) embryos. In control embryos (n=12) the caudal motoneuron (CaP) extends axons ventrally through the somite (red outline). Rostral motoneurons (RoPs) extend axons ventrally to the level of the horizontal myoseptum and then move anteriorly (yellow outline, Figure 4M). Lamc3 knockdown embryos (n=12) show that although CaPs migrate normally, they branch more frequently ( Figure 4N). Furthermore, the number of RoPs at the horizontal myoseptum is significantly reduced (p<0.0001, Unpaired Student’s t-test) and additional rounded *gfap*:gfp expressing cells are found in the trunk of the embryos ( Figure 4N,O). The specification of HB9 positive cells and CaPs suggests that neural tube development is not affected by Lamc3 knockdown and that Lamc3 is not necessary for motoneuron specification, but may be required for regulating morphology, polarity or axon migration.

## Discussion

### Ectopic *prox1a*-expressing cells at the horizontal myoseptum

*Prox1a* is expressed by a number of tissues including slow-twitch muscle, motoneurons, and thoracic duct ( Dunworth *et al*., 2014). We were unable to determine the nature of the additional *prox1a*-expressing cells seen in Lamc3 knockdown embryos ( Figure 4D), but bearing in mind their location we suggest these represent muscle pioneers. These data indicate there may be a delay in muscle pioneer differentiation into slow twitch muscle, or else indicate that several cell types at the horizontal myoseptum are affected by Lamc3 knockdown. This requires further investigation.

### Study of Netrin-1 in Lamc3 knockdown embryos

*Netrin-1a* expression was up-regulated in the neural tube of Lamc3 knockdown embryos. Although this might explain the increase of Netrin-1 protein observed in transverse sections, the way in which Netrin-1a is regulated by Lamc3 remains unclear. Western blot analysis of zebrafish proteins indicates that the antibody used is specific to Netrin-1, and immunostaining appears to recapitulate the Netrin-1a *in situ* hybridisation expression pattern in muscle pioneer cells. However, we were unable confidently to distinguish between Netrin-1a and Netrin-1b. We suggest that further analyses using this antibody might benefit from immunofluorescence staining on Netrin-1a morphants or western blot analyses of protein extracted from Netrin-1a knockdown embryos.

### Use of morpholino oligonucleotides and CRISPR/Cas9 technologies

In recent years several technical concerns have been raised suggesting that morpholino oligonucleotides cause phenotypes that are not recapitulated in genetic mutants ( Kok *et al*., 2015; Novodvorsky *et al*., 2015). Indeed, our own group has also shown discrepancies between the phenotypes of morphants and mutants ( Eve *et al*., 2017; Place & Smith, 2017). Such variation between phenotypes might be caused by genetic compensation, maternal mRNA and protein contributions in mutant embryos, or nonspecific off-target effects of morpholino injection ( Kok *et al*., 2015; Maurya *et al*., 2013; Rossi *et al*., 2015). We were keen to generate a *lamc3* mutant line with which to explore Lamc3 function and were disappointed that our initial attempts suggested that such animals would not be viable. Although the oedema seen in *lamc3* CRISPR/Cas9 injected F0 larvae was in tissues which endogenously express *lamc3* (specifically the gut, eye, and otic vesicle) it remains possible that this is a nonspecific effect rather than a phenotype of γ3-deficiency, because no oedema was observed in Lamc3 SBMO embryos at 5 dpf. Although further attempts to generate a *lamc3* genetic line were not possible within the scope of this work, we would encourage attempts to generate a *lamc3* loss-of-function mutant in any further study of Lamc3, as suggested by the zebrafish community ( Stainier *et al*., 2017). We suggest that viability might be improved by targeting a different region of the gene or functional domain of the protein.

Because we were unable to generate a mutant line, genetic investigations were limited to F0 CRISPR/Cas9 injected embryos, which phenocopy the PAC defects seen in MO knockdown embryos. Although previous studies have used F0 CRISPR/Cas9 injections to complement morpholino knockdown data, this technology is not without limitations ( Jao *et al*., 2013; Kim *et al*., 2017; Sharma *et al*., 2016). First, cells in such embryos are mosaic and may carry different types of mutation, or indeed no mutation at all. While we could show that high levels of mutagenesis in sgRNA1 injected embryos correlated with the presence of the PAC phenotype we cannot be confident that all cells in the embryo carried the same mutations, and nor whether these were true loss-of-function mutations. Therefore, the interpretation of these results in whole embryos is challenging. Second, introduction of sgRNAs might cause off-target effects like those seen with morpholinos. We did not see any PAC defects using sgRNA2 that proved ineffective at inducing mutations, nor did we see significantly more defects when injecting sgRNA1 without Cas9, supporting the contention that PAC defects were not off-target effects.

### Lamc3 and neuronal guidance

We observed that rostral primary motoneurons failed to migrate properly and caudal primary motoneurons had ectopic branches in Lamc3 knockdown embryos. This suggests that laminin γ3 is required for motoneuron axon guidance. If this is the case, the observed defects in parachordal chain development are likely to be a consequence of an earlier neuronal defect ( Lim *et al*., 2011). A role for LAMC3 in neuronal migration has already been described in mammals. In mice, double knock out of *Lamb2* and *Lamc3* affected the migration of astrocytes and dopamingeric neurons in the developing retina, where *Lamc3* is highly expressed ( Dénes *et al*., 2007; Gnanaguru *et al*., 2013; Li *et al*., 2012; Pinzón-Duarte *et al*., 2010). Although this is a different system from the zebrafish trunk, these data might suggest a conserved role for γ3 in vertebrate neuronal guidance, warranting further exploration.

The mechanism by which Lamc3 might regulate axon migration also remains unclear. Laminin γ3 might repel axon growth cones directly as described previously for laminin-1 ( Höpker *et al*., 1999; Ratcliffe *et al*., 2008). Excessive motoneuron branching is also observed when guidance of the axon growth cones is disturbed, such as in N-cadherin or PlexinA3 knockdown embryos ( Brusés, 2011; Feldner *et al*., 2007). Furthermore, the *lama1* zebrafish mutant, *bashful*, also has axon guidance defects including branched caudal motoneurons and the absence of the rostral primary motoneuron, which might indicate laminin α1 and laminin γ3 form a complex for axon guidance ( Paulus & Halloran, 2006). Amino acid sequence analysis showed that zebrafish γ3 lacks a glutamic acid residue required for binding integrins ( Ido *et al*., 2008). This suggests that γ3 might not be able to interact with cells directly, although γ3 may still bind through an alternative domain. Indeed, studies suggest that laminins containing γ3 bind α6β1 integrins in mouse testes ( Yan & Cheng, 2006).

In zebrafish, laminin γ3 was found at the periphery of the embryo at the dorsal neural plate, horizontal myoseptum, and ventral vessels. Netrin-1 staining identified protein in these same regions, with the exception of the vasculature. However, in Lamc3 SBMO embryos Netrin-1 was detected throughout, which suggests γ3 might be required for the localisation of Netrin-1 to specific regions. Netrins and laminins have been shown to interact in mice ( Schneiders *et al*., 2007). It is possible that γ3 binds soluble Netrin-1 at the horizontal myoseptum to form a haptotactic substrate for the local guidance of primary motoneurons. Indeed, this was recently shown to be the mechanism of neuronal guidance in the mouse neural tube ( Dominici *et al*., 2017; Varadarajan *et al*., 2017). More work is required to determine whether this is the mechanism for γ3 function in the zebrafish.

## Conclusion

We show that zebrafish parachordal chain development is affected by loss of Lamc3, whether mediated by morpholino knockdown or by CRISPR/Cas9 mutagenesis in F0 embryos. We find that muscle pioneer cells are specified and express *netrin-1a* in the absence of Lamc3. However, under knockdown conditions the localisation of Netrin-1 protein extends beyond its domain of the horizontal myoseptum, perhaps because it is normally anchored by γ3. We also observe abnormal migration and morphology of trunk motoneurons, which might be explained by mis-localisation of Nerin-1. We suggest the parachordal chain phenotype in γ3-deficient embryos is a consequence of the failure of rostral motoneurons to migrate at the horizontal myoseptum.

## Data availability

All datasets underlying the results presented in this manuscript are available from OSF: http://doi.org/10.17605/OSF.IO/C5BKG ( Eve, 2017). 

**File 1 - MUSCLE alignments of LAMC1, LAMC2 and LAMC3 proteins.** Raw data generated from MUSCLE alignments of laminin gamma (LAMC) proteins from *Nematostella vectensis*, *Mus musculus*, *Danio rerio* and *Homo sapiens*.

**File 2 - Trimmed alignments of LAMC1, LAMC2 and LAMC3 proteins for phylogenetic tree building**. Raw data used for generating phylogenetic trees, gaps and areas of low conservation of the aligned amino acid sequences from File 1 were trimmed by eye.

**File 3 - Raw quantitative RT-PCR data (Cp-values).** Duplicate raw Cp-values from qPCR analysis of cDNA synthesised from GFP FACS-sorted Tg( *fli1a*:egfp) transgenic zebrafish. GFP-ve, ** cells that did not express *GFP*; GFP+ve, *GFP* expressing cells; No RTase, No reverse transcription of RNA control. Dilution curve and negative (water) controls as labelled.

**File 4 - Number of intersegmental vessels and parachordal chains at 48 hpf.** Counting of vessels in uninjected, standard control morpholino injected, and Lamc3 splice-blocking morpholino (SBMO) and translation-blocking morpholino (TBMO) injected embryos. Hpf, hours post fertilisation.

**File 5 - Number of intersegmental vessels and parachordal chains at 72 hpf.** Counting of vessels in uninjected, standard control morpholino injected and Lamc3 splice-blocking morpholino (SBMO) and translation-blocking morpholino (TBMO) injected Tg( *fli1a:*egfp) embryos. Hpf, hours post fertilisation.

**File 6 - Number of hemisegments with thoracic duct at 5 dpf.** Counting of thoracic duct vessels in standard control morpholino injected and Lamc3 splice-blocking morpholino (SBMO) injected Tg( *kdrl*:gfp, *prox1a*:rfp) embryos. Dpf, days post fertilisation.

**File 7 - Number of HB9 cells at 2 dpf.** Counting of HB9 immunostained cells in standard control morpholino injected and Lamc3 splice-blocking morpholino (SBMO) injected embryos. Dpf, days post fertilisation.

**File 8 - Number of caudal and rostral motoneurons at 2 dpf.** Counting of caudal and rostral motoneurons in standard control morpholino injected and Lamc3 splice-blocking morpholino (SBMO) injected Tg( *gfap*:gfp) embryos. Dpf, days post fertilisation.

**File 9 - Raw data of Western blot gel used in Figure 4I.**

## Supplementary material

**Figure S1.** Examples of image manipulation from raw data (original images) as labelled.

**Figure S2.** Overexpression of mouse Lamc3 mRNA. ( **A**– **C**) Anti-LAMC3 immunostaining in the dissected trunks of 24 hpf embryos either uninjected ( **A**), injected with 1 nl of 200 ng/μl of mouse Lamc3 (mLamc3) mRNA ( **B**) or injected with 1 nl of 400 ng/μl of mouse Lamc3 mRNA shows increase of antibody staining at the horizontal myoseptum, dorsal plate of the neural tube and cloaca. ( **D**– **F**) Imaging of the trunk vasculature in Tg( *fli1a*:egfp) 50 hpf embryos either uninjected ( **D**), injected with 1 nl of 200 ng/μl of mouse Lamc3 mRNA ( **E**) or injected with 1 nl of 400 ng/μl of mouse Lamc3 mRNA ( **F**). A, anterior; D, dorsal; hpf, hours post fertilisation. Scale bars: 100 μm.

**Figure S3.** p53 and Lamc3 translation blocking morpholino (TBMO) injections. ( **A**) 48 hours post fertilisation (hpf) embryo injected with standard control morpholino. ( **B**) 48 hpf embryo injected with Lamc3 splice-blocking morpholino (SBMO) without p53 morpholino causes reduced eye size and oedema in the head. ( **C**) Tg( *fli1a*:egfp) embryo at 72 hpf injected with Lamc3 TBMO and p53 morpholino shows loss of the parachordal chain.

**Figure S4.** sgRNA injection does not affect parachordal chain (PAC) development. ( **A**) In 50 hpf Tg( *fli1a*:egfp) embryos injected with 1nl of 12–15 ng/μl of sgRNA1 failure of PAC development (red asterisk) is observed in fewer than 5 hemisegments at the yolk extension per embryo (48/51). ( **B**) A third of embryos injected with sgRNA1 and Cas9 mRNA have perturbed development of the PAC in 5 or more hemisegments at the level of the yolk extension (16/47). A, anterior; D, dorsal; hpf, hours post fertilisation.
