# Supplementary figures and images for: Knockdown of Laminin gamma-3 (Lamc3) impairs motoneuron guidance in the zebrafish embryo
Source: Wellcome Open Res. 2017 Nov 16;2:111. [Version 1] doi: 10.12688/wellcomeopenres.12394.1 (PMC5785718; doi:10.12688/wellcomeopenres.12394.1)

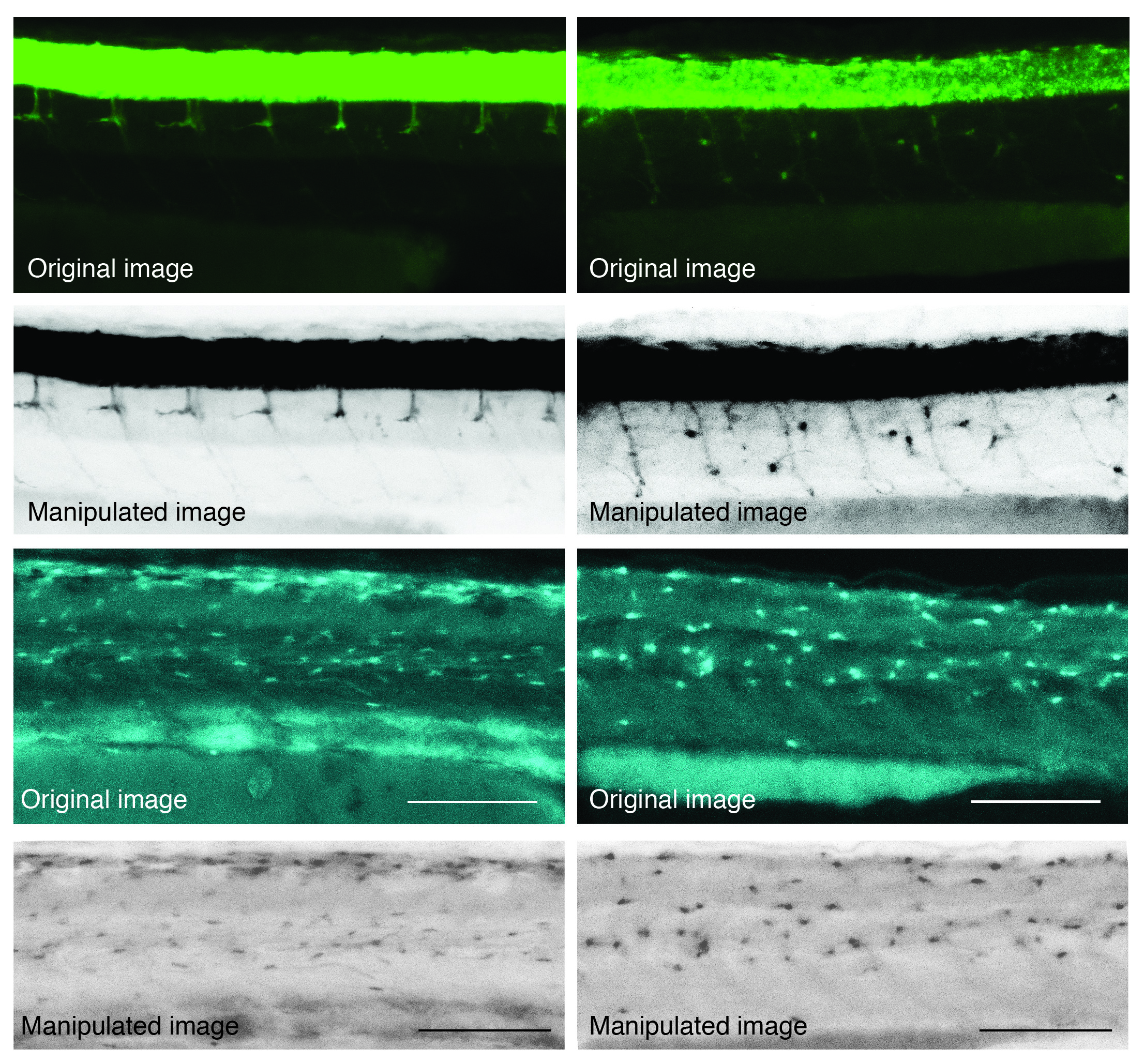

Supplement: Supplementary file 1 [file wellcomeopenres-2-13423-s0000.tgz › 42a4cb23-0903-4eed-a11b-dc90177e1d36.jpg]

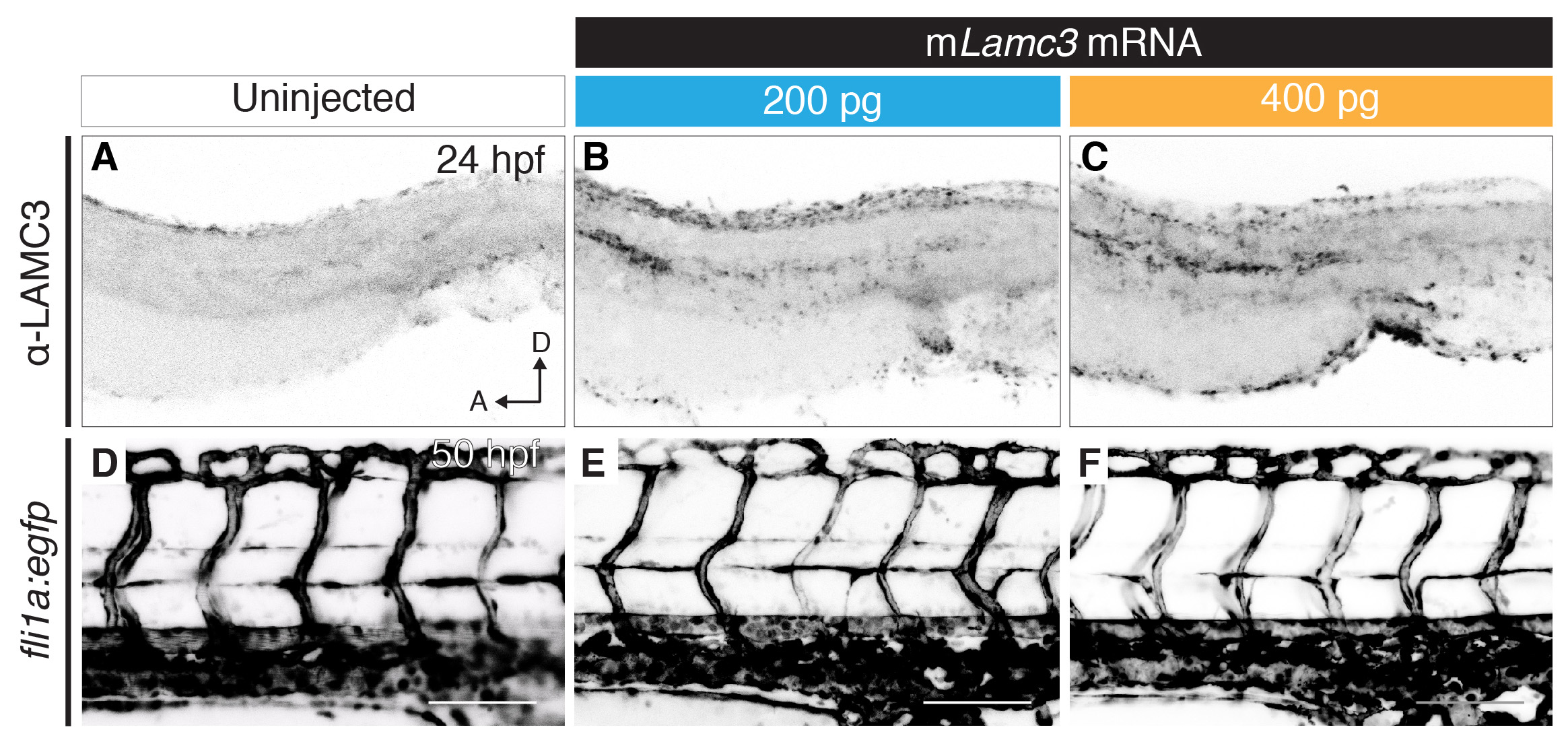

Supplement: Supplementary file 2 [file wellcomeopenres-2-13423-s0001.tgz › e8a653ee-afb9-4775-8a57-49dbb97431ba.tif]

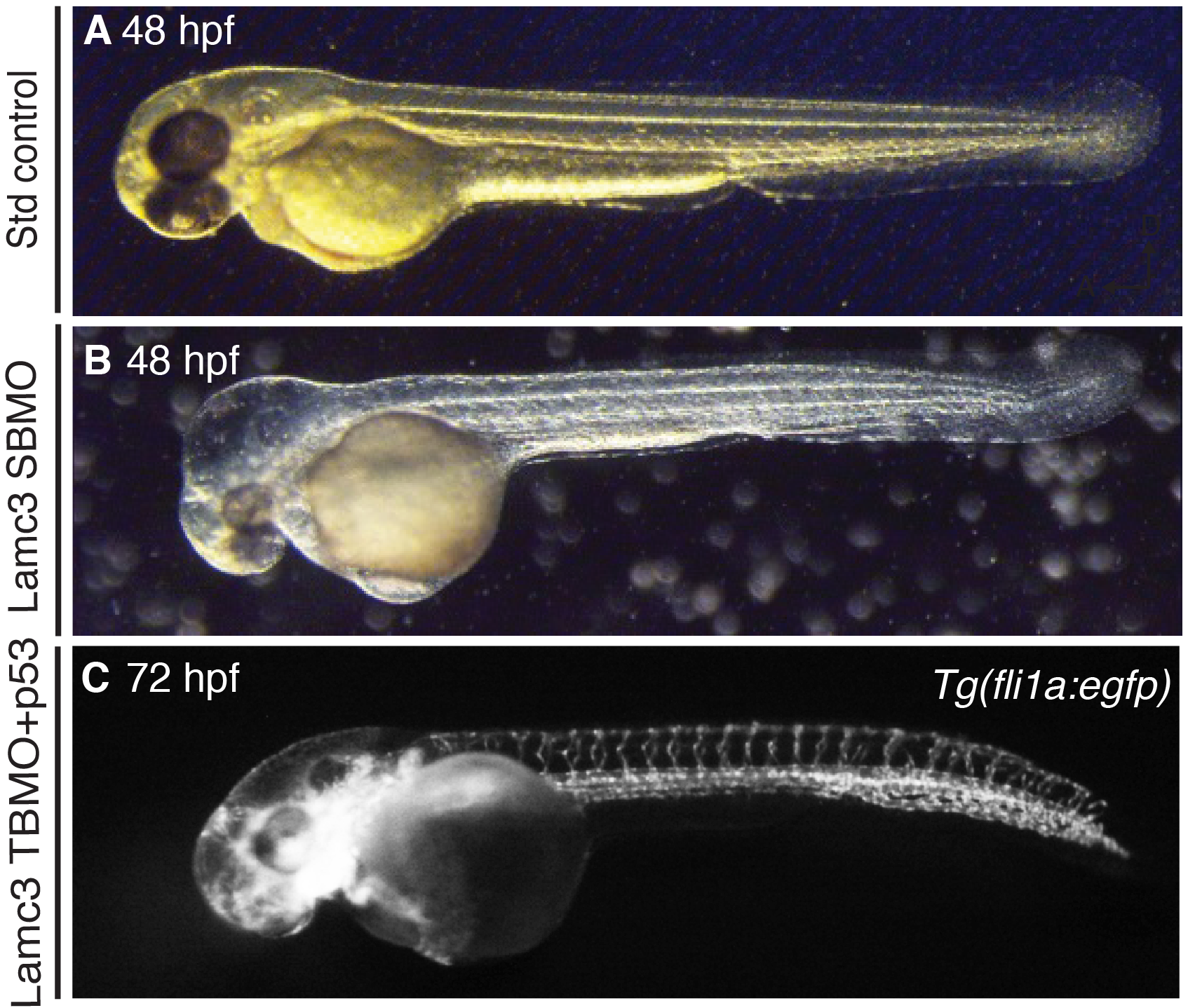

Supplement: Supplementary file 3 [file wellcomeopenres-2-13423-s0002.tgz › 17086b00-564c-49d1-a9a3-ce915efbf2c1.png]

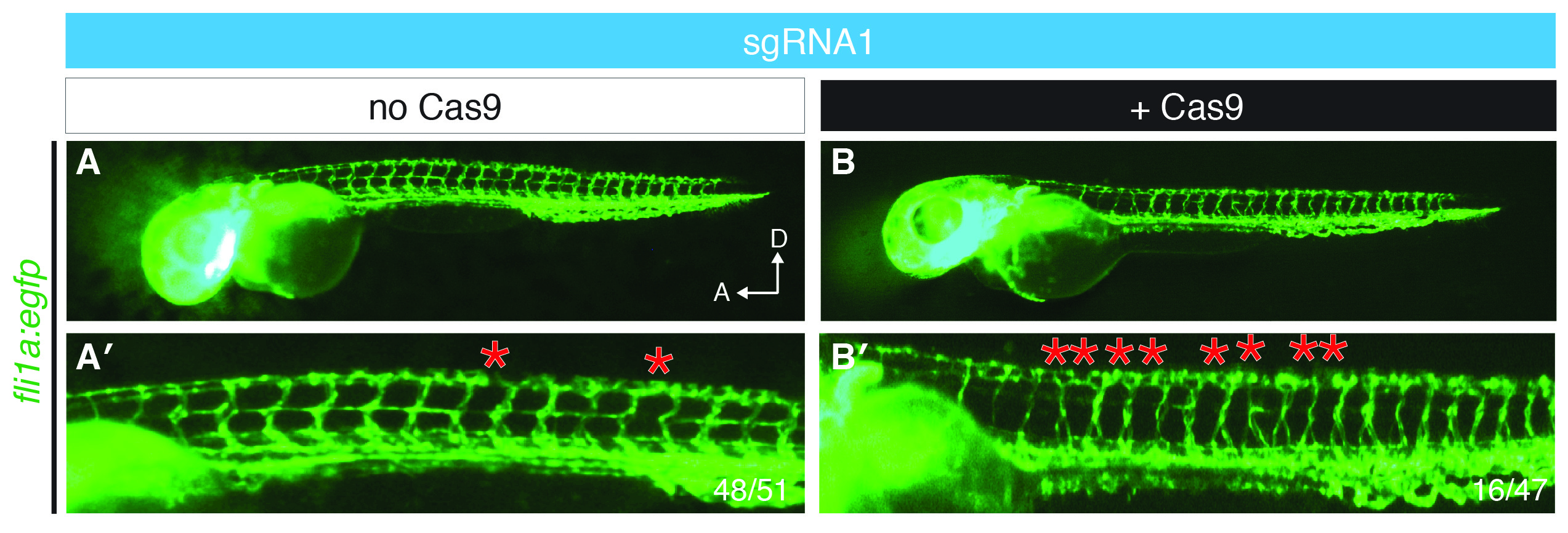

Supplement: Supplementary file 4 [file wellcomeopenres-2-13423-s0003.tgz › bacb6d16-4e4d-4dd3-8aa5-8068a912aa51.jpg]
