# Supplementary material for: Knockdown of Laminin gamma-3 (Lamc3) impairs motoneuron guidance in the zebrafish embryo
Source: Wellcome Open Res. 2017 Nov 16;2:111. [Version 1] doi: 10.12688/wellcomeopenres.12394.1 (PMC5785718; doi:10.12688/wellcomeopenres.12394.1)
Supplement: Peer review history

# Peer review: Referee response for version 1

**DOI:** 10.21956/wellcomeopenres.13423.r29423

**Published:** 2018-01-26

**Authors:** Thomas A. Hawkins

**Affiliations:** 1 Division of Biosciences, Cell and Developmental Biology, University College London, London, UK

This is a well constructed and clearly written manuscript.  The authors present a straightforward story of a knockdown of laminin 3 (Lamc3) and the consequence of this on parachordal chain and motorneuron development.  The knockdown of Lamc3 was achieved using morpholino oligonucleotide injection that was validated by transient, mosaic genetic lesions in F0 embryos using using CRISPR/Cas9.  They indicate that upon reduced lamc3 expression, the parachordal chain is lost and that motorneuron morphology is perturbed; the former defect is suggested to be the consequence of the latter, consistent with earlier findings.   Abnormal midline development evidenced by altered netrin expression and changes in prox1a transgene expression as a consequence of the loss of lamc3 is cited as a probable cause of the defects.

 The experimental procedures admirably well presented: they are thoroughly described, and they seem to have been carried out carefully.  The data presented are of a high quality.  The authors are sensibly cautious and measured in the language employed to describe their results and to conclude from them.  Their findings do throw up several further questions and many of these are discussed by the authors.  I might normally ask for more experiments to develop the study more but I recognise that the reviewer guidelines are limited to an analysis of the quality of the work presented; discussion of the further questions would seem to suffice in this context.  Nevertheless, I do have a few reservations about the study, as it is presented, these can probably be addressed through adjustment of the manuscript.  The main issues are listed below, followed by some smaller items that would improve/correct the manuscript prior to formal publication.

 Larger issues

  

1. Because the study heavily relies upon morpholino use, it is imperative to carefully validate the efficacy of the reagents employed.  The authors employ several approaches to this problem, including the use of both splice- and translation-blocking morpholinos, immunohistochemistry against the target protein as well as the replication of the findings using transient CRIPSR/Cas9 injection. This is good and refreshing when compared with other published data employing morpholinos.  I believe the specificity and efficacy of the morpholino employed is reasonably sure.  However, I was a bit surprised to see that the authors gave up attempting the generation of a full mutant line after apparently only attempting to raise 72 F0 embryos/larvae with only 12 making it to adulthood for genomic testing.  (As an aside here the authors do not make clear from what tissue they tested the 12 adults, presumably sample embryos from crosses of the individuals were used? The germ line is obviously the crucial place to test for a heritable mutation.  This should be clarified).  With the apparently reliable restriction digest assay that the authors have already generated I would have thought it were feasible to either titrate down the dose of the Cas9/sgRNA injections sufficiently, or use the less efficient sgRNA so that there was less mutation load to allow more fish to survive to adulthood for germ line genomic lesion screening.  This is certainly a time-consuming undertaking because of generation time, and that may be the problem here but the authors should be honest about that.  The morpholino data is certainly convincing as it stands but, as acknowledged by the authors in the discussion, consensus in the community is that bona fide genetic mutants are a ‘gold standard’ for examination of gene function, particularly also in concert with morpholino use. I feel that the presented reasons for abandoning efforts to obtain a lamc3 mutant are not really sufficient.  I recommend a change to this section to acknowledge it may be possible in the future to generate a mutant using the same reagents employed for ‘morpholino validation’ here.
2. It is not clear whether the authors were able to correlate more severe genetic lesions using CRISPR/Cas9 and sgRNAs with more severe PAC defects.  It seems logical to me that this would be done but the two results are presented separately.  Was this carried out?  Also, in other studies employing F0 mutagenesis, experimenters have multiplexed their sgRNAs to increase the chance of lesion.  Did the authors try this approach?
3. In Fig.4G’ there is an abrupt drop off in the NTN1 staining on the left hand side of the sectioned embryo, when passing from dorsal to ventral there is almost a line apparent, below which the expression is much lower compared to the right hand side.  What is the cause of this? Was it consistently seen? Perhaps it is a sectioning artifact? Despite this it is clear that the morphant (Panel H’) staining is more extensive, the difference between sides in the Std control section is something that stands out. Please comment on this.

 

 Smaller issues

  

1. A little more detail on the qRT-PCR experiment would be good eg. How was the FACs undertaken?  How was the RNA isolated? Were the primer efficiencies determined? Are the n numbers in the panel Fig1D replications of the whole experiment (FACs sorting and qRT-PCR) or just the qRT-PCR? I realise it is a negative result but the detail helps with interpretation.
2. In the methods section, under the probe synthesis and *in situ* hybridisation heading, the manscript states that the T7 promoter sequence is underlined and SP6 sequence is in italics, neither can be seen in the primer sequences as presented in the version I see online and in PDF.
3. Figure 3 issues: a) The n-numbers for the data in panel E would be useful to present in the panel or at least in the legend.  I can see they are in the body but it is much easier for the reader to see them either in legend or panel itself; b) In Fig 3J the blue box above the gel image says “Surviving F0 SG RNA *embryos …”* where the legend states *adult* fish; c) The ordering of the panel labelling in fig. 3 is rather odd: Panels A-G are labelled left to right by row and H-J are by column, it makes it hard to read; d) The picture of the three embryos dorsally mounted in the bottom right of figure 3 does not have a panel label or a corresponding legend; e) In my opinion, the “U” in Panel G that denotes undigested would be better described in the section of the legend about this panel rather than at the end of the legend; f) There is no “U” sample in panel H, why? This is a different assay and it would be useful for interpretation.
4. I believe that the results section entitled “Lamc3 is required for parachordal chain (PAC) formation” was intended to go after the “Lamc3 is required for parachordal chain (PAC) formation”.  It seems like the more logical order.  This is especially true regarding the p53 MO use.  The order it is written currently it is ambiguous whether the experiments described in the earlier section employed co-injection of p53 MO or not.  I suspect an editing error but, either way, the authors should make clear which experiments employed p53 MO in addition to the various other morpholinos (including Std control).

 

 If the authors are able to address the points above I would be happy for the paper to be formally published.  As I hope is clear, the changes are mainly possible to be dealt with through changes in the text.  Some of the points I raise are open to debate and I’m happy to engage in this, if necessary.

I have read this submission. I believe that I have an appropriate level of expertise to confirm that it is of an acceptable scientific standard, however I have significant reservations, as outlined above.

# Peer review: Referee response for version 1

**DOI:** 10.21956/wellcomeopenres.13423.r29476

**Published:** 2018-01-24

**Authors:** Nicola Facchinello

**Affiliations:** 1 Department of Biology, University of Padova, Padova, Italy

In this paper, the Authors used a morpholino and CRISPR/Cas9 approach and they find a  role of laminin γ3 in motoneuron guidance. The experiments are technically sound and the results are clear. The images are of high quality and the quantification data are well-described, including numbers of animals and numbers of experiments.  The manuscript is well written and may be recommended for indexing.

 For revision, the following changes are recommended:

 Fig1 (Panel D) Were sufficient animals analysed to conclude that no difference exists? It would be interesting if the authors presented a double IF to show if there is a colocalization of Lamc3 (red) and fli1a  (anti-GFP) in some vessels.

 Fig2. To assess the morpholino efficacy, lam3c specific RT-PCR reactions could be performed on RNA populations or check the decrease of protein level by western blot?

 Did you try also a mismatch MO instead of std control?

 Fig3 (Panel A) Did you check for sgRNA1 the decrease of mRNA or protein level for Lam3c in the mutant with the phenotype?

 A suggestion could be the genotyping of mutations in F0 using heteroduplex mobility assay (HMA) ^1^. In this way you can identify some mutation that could not be identified by digestion. To try to obtained a stable line in the CRISPR/Cas9 approach, you can try to decrease the concentration and nanoliters inject of sgRNA1 due to its toxicity or too high efficiency.

I have read this submission. I believe that I have an appropriate level of expertise to confirm that it is of an acceptable scientific standard.

## References

[1] ZhuXXuYYuSLuLDingMChengJSongGGaoXYaoLFanDMengSZhangXHuSTianY: An efficient genotyping method for genome-modified animals and human cells generated with CRISPR/Cas9 system. *Sci Rep*.2014;4: 10.1038/srep06420 6420 10.1038/srep06420 25236476PMC4168274

# Peer review: Referee response for version 1

**DOI:** 10.21956/wellcomeopenres.13423.r29478

**Published:** 2018-01-22

**Authors:** Dong Liu

**Affiliations:** 1 Co-Innovation Center of Neuroregeneration, Jiangsu Key Laboratory of Neuroregeneration, Nantong University, Nantong, China

The authors provided with evidences that lamc3 was enriched in endothelial cells as a regulator of retinal neuronal guidance. However, it has not been investigated on zebrafish model. The authors performed Lamc3 knockdown and knockout using Mo and CRISPR/Cas9 approaches during zebrafish development. It was revealed that Lamc3 deficiency disturbed the formation of the PAC, the thoracic duct, and axonal guidance of rostral primary moto neuron which might be caused by up-regulation of netrin-1a in the neural tube, but not sprouting of the cardinal vein. The manuscript was well written and the figures were well presented. However, I have some questions that need to be addressed:

1. In the results section, the authors discribed that LAMC3 is most closely related to LAMC1. Whether LAMC1 were closer to the clade of LAMC2 based on Fig.1B. ?
2. In Fig.1G-H, it will be more convincing to colocalize the expression of LAMC3 and endothelial cell specific gene, such as *kdrl*, using immunostaining or the transgenic lines which express florescent proteins in endothelial cells.
3. In the Figure1, Is the legend (I) indicating the panel (H’)?
4. The content of figure S4 was similar with figure3B-D. What is the difference between them?
5. The authors showed that lamc3 loss-of-function impaired the formation of parachordal chain (PAC) and thoracic duct. To confirm the phenotype was caused specifically by loss of lamc3, I suggest the authors to examine whether lamc3 mRNA could rescue the phenotypes in morphants and mutants.
6. Since the impaired blood flow affects the formation of parachordal chain (PAC) and thoracic duct in zebrafish. Whether the phenotype of lamc3 deficient embryos was caused by blood flow defect as a secondary effect needs to be seriously checked.
7. line is often employed in the study of astrocytes. Why did the authors chose the *Tg(gfap:gfp)* line to study motoneuron development instead of *Tg(mnx1:EGFP)*?
8. Motoneurons are essential for vascular pathfinding. Were the parachordal chain (PAC) defects were caused by primary motoneurons malformation?
9. In figure 4F, expression domain of netrin-1a was increased in the neural tube, the netrin1a signals in horizontal myoseptum and the whole embryo were stronger too. In contrast, the authors found that the netrin-1 protein was reduced in all Lamc3 knockdown embryos at the level of the horizontal myoseptum compared with controls, the authors should give some interpretation.
10. Results that netrin expression was affected by LAMC3 knockdown are interesting. I suggest the authors add the relevant discussion, for example the potential molecular mechanism.

I have read this submission. I believe that I have an appropriate level of expertise to confirm that it is of an acceptable scientific standard.

# Peer review: Referee response for version 1

**DOI:** 10.21956/wellcomeopenres.13423.r28075

**Published:** 2017-12-13

**Authors:** Timothy J.A. Chico

**Affiliations:** 1 Department of Infection, Immunity & Cardiovascular Disease, Medical School, The Bateson Centre for Lifecourse Biology, University of Sheffield, Sheffield, UK

SUMMARY

 I enjoyed reading this paper, which was conducted technically well and presented in a very accessible and thorough manner. The authors are sensibly cautious about the conclusions that they draw. I  wholeheartedly believe that the huge amount of unpublished morpholino data, were it available to the community, would be of great value even with the difficulty in being certain that all the described effects are specific so am very keen that this paper, and the hundreds like it that I suspect could be written, be disseminated.

 I make some general comments below, but the main point that I think should be kept in mind when interpreting this paper is that non-specific effects on cardiac output induced by the morpholino or the CRISPR gRNA would be expected to induce the same reduction in lymphatic formation; unless the authors revise the manuscript to include an assessment of cardiac output and blood flow then I would be a little uncomfortable assuming that these effects have much to do with LAMC3 directly. It would be reasonable however, to acknowledge this and other potential confounders a little more clearly in the results and discussion if it is not possible at this stage to examine the effects of the genetic manipulations on blood flow.

 The immunostaining against Laminin 3 (Fig 1) is nice but without more validation that it binds selectively to zebrafish LAMC3 (such as a western blot) it is hard to be certain that the immunostaining is specific to this isoform of laminin, especially with a cross-species antibody. The overexpression study in the supplemental data does show that there is increased antibody binding to mouse LAMC3 when overexpressed but does not rule out it binding to other laminins or non-specifically, nor does it confirm it binds to zebrafish LAMC3. I would therefore consider the protein expression data to be likely correct but not completely validated. However, the reduction of staining in the LAMC3 morphants makes it likely that the antibody binds to the MO target; it just cannot be concluded that it doesn't bind to anything else!

 Figure 2 shows nicely that the LAMC3 morpholino prevents lymphatic formation. However, a similar effect is seen if blood flow is prevented in embryos, for example by troponin knockdown. To be certain that this does not explain the observed phenotype, it would be necessary to show that the morpholino does not alter cardiac output and blood flow in the dose used.

 The CRISPR/Cas9 methods indicate that some embryos were injected with gRNAs alone (which is an appropriate control) but the results don't show these data; they only show Cas9 alone as a control. My own group has found that some gRNAs at high doses do induce toxicity and these data should be shown or discussed. I'm not convinced that the different effects of the second guide RNA exclude off target toxicity from a different guide; we see varying effects with different guide sequences.

 Although the LAMC3 crispants show similar defects in lymphatic development as the morphants, again this could be secondary to alterations in blood flow which would need to be examined to exclude such a confounding effect.

 The authors do seem to have encountered a problem with the CRISPR/Cas9 system, in that they have found a really efficient guideRNA that cuts bi-allelically and so induces a lethal phenotype that means a heterozygous founder cannot be identified. It would have made more sense to raise the embryos injected with the second, less efficient guide as this does seem to induce mosaic/heterozygous mutations more compatible with finding a founder adult.

 The alteration of netrin expression induced by LAMC3 knockdown is interesting, but would be strengthened by quantification (in addition to the usual 12/13 type annotation which cannot be confirmed by a reviewer). For example, if the expression domains are quantified blindedly, is there a statistically significant alteration? It seems this would be very achievable and would make the conclusions far more solid. In passing, no mention of blinding during analysis is made in the methods, and it would be important to know if when the quantification was performed in for example Figure 2 that the observer was blinded to group allocation.

 Minor points

 Table 1 refers to concentrations of morpholinos but these are doses per embryo.

I have read this submission. I believe that I have an appropriate level of expertise to confirm that it is of an acceptable scientific standard, however I have significant reservations, as outlined above.
